# Diagnostic accuracy, incremental yield and prognostic value of Determine TB-LAM for routine diagnostic testing for tuberculosis in HIV-infected patients requiring acute hospital admission in South Africa: a prospective cohort

**DOI:** 10.1186/s12916-017-0822-8

**Published:** 2017-03-21

**Authors:** Stephen D. Lawn, Andrew D. Kerkhoff, Rosie Burton, Charlotte Schutz, Andrew Boulle, Monica Vogt, Ankur Gupta-Wright, Mark P. Nicol, Graeme Meintjes

**Affiliations:** 1 Department of Clinical Research, Faculty of Infectious and Tropical Diseases, London School of Hygiene and Tropical Medicine, London, UK; 2 The Desmond Tutu HIV Centre, Institute of Infectious Disease and Molecular Medicine, Faculty of Health Sciences, University of Cape Town, Cape Town, South Africa; 3 Department of Medicine, Faculty of Health Sciences, University of Cape Town, Cape Town, South Africa; 4 Department of Medicine, University of California San Francisco School of Medicine, San Francisco, CA USA; 5 GF Jooste Hospital, Manenberg, Cape Town, South Africa; 6 Khayelitsha District Hospital, Cape Town, South Africa; 7 Clinical Infectious Diseases Research Initiative, Institute of Infectious Disease and Molecular Medicine, University of Cape Town, Cape Town, South Africa; 8 School of Public Health and Family Medicine, Faculty of Health Sciences, University of Cape Town, Cape Town, South Africa; 9 Division of Medical Microbiology and Institute for Infectious Diseases and Molecular Medicine, Faculty of Health Sciences, University of Cape Town, Cape Town, South Africa; 10 National Health Laboratory Service, Groote Schuur Hospital, Cape Town, South Africa

**Keywords:** Tuberculosis, HIV, Diagnosis, Urine, Xpert, Lipoarabinomannan, LAM, Africa, Screening, Hospital, Determine TB-LAM

## Abstract

**Background:**

We previously reported that one-third of HIV-positive adults requiring medical admission to a South African district hospital had laboratory-confirmed tuberculosis (TB) and that almost two-thirds of cases could be rapidly diagnosed using Xpert MTB/RIF-testing of concentrated urine samples obtained on the first day of admission. Implementation of urine-based, routine, point-of-care TB screening is an attractive intervention that might be facilitated by use of a simple, low-cost diagnostic tool, such as the Determine TB-LAM lateral-flow rapid test for HIV-associated TB.

**Methods:**

Sputum, urine and blood samples were systematically obtained from unselected HIV-positive adults within 24 hours of admission to a South African township hospital. Additional clinical samples were obtained during hospitalization as clinically indicated. TB was defined by the detection of *Mycobacterium tuberculosis* in any sample using Xpert MTB/RIF or liquid culture. The diagnostic yield, accuracy and prognostic value of urine-lipoarabinomannan (LAM) testing were determined, but urine-LAM results did not inform treatment decisions.

**Results:**

Consecutive HIV-positive adult acute medical admissions not already receiving TB treatment (*n* = 427) were enrolled regardless of clinical presentation or symptoms. TB was diagnosed in 139 patients (TB prevalence 32.6%; median CD4 count 80 cells/μL). In the first 24 hours of admission, sputum (spot and/or induced) samples were obtained from 37.0% of patients and urine samples from 99.5% of patients (*P* < 0.001). The diagnostic yields from these specimens were 19.4% (*n* = 27/139) for sputum-microscopy, 26.6% (*n* = 37/139) for sputum-Xpert, 38.1% (*n* = 53/139) for urine-LAM and 52.5% (*n* = 73/139) for sputum-Xpert/urine-LAM combined (*P* < 0.01). Corresponding yields among patients with CD4 counts <100 cells/μL were 18.9%, 24.3%, 55.4% and 63.5%, respectively (*P* < 0.01). The diagnostic yield of urine-LAM was unrelated to respiratory symptoms, and LAM assay specificity (using a grade-2 cut-off) was 98.9% (274/277; 95% confidence interval [CI] 96.9–99.8). Among TB cases, positive urine-LAM status was strongly associated with mortality at 90 days (adjusted hazard ratio 4.20; 95% CI 1.50–11.75).

**Conclusions:**

Routine testing for TB in newly admitted HIV-positive adults using Determine TB-LAM to test urine provides major incremental diagnostic yield with very high specificity when used in combination with sputum testing and has important utility among those without respiratory TB symptoms and/or unable to produce sputum. The assay also rapidly identifies individuals with a poor prognosis.

**Electronic supplementary material:**

The online version of this article (doi:10.1186/s12916-017-0822-8) contains supplementary material, which is available to authorized users.

## Background

Tuberculosis (TB) is the leading cause of HIV/AIDS-related mortality globally, accounting for an estimated 0.4 million HIV-related deaths worldwide in 2015 [1, 2]. A large majority of these deaths occur in sub-Saharan Africa. Post-mortem studies conducted in African hospitals over the past 20 years have revealed that between 32% and 67% (pooled summary prevalence estimate 39.7%; 95% confidence interval [CI] 32.4–47.0) of HIV-positive adult patients had evidence of TB disease at autopsy [3–5] and that this was the primary cause of death in over 90% of these cases [6]. Disease was frequently disseminated and approximately half of these cases remained undetected prior to death [6]. These findings suggest the need for new approaches to routine diagnostic screening for TB in HIV-infected patients newly admitted to hospital in resource-limited settings.

Using an intensive clinical sampling approach, we have previously demonstrated that one in three unselected HIV-infected acute medical adult admissions to a South African township district hospital had TB that could be diagnosed microbiologically [7]. Presentation was so non-specific that neither symptom screening nor risk profiling were sufficient to reliably identify a low-risk group that did not warrant further laboratory investigation for TB. We therefore concluded that to maximize TB case ascertainment, all HIV-infected patients newly admitted to hospital in settings with high TB burden should be investigated for TB using microbiological tests regardless of their clinical presentation. Unfortunately, laboratory capacity is often severely limited in resource-limited settings. Moreover, it is frequently challenging to obtain sputum samples from sick medical inpatients, even from those suspected of having pulmonary TB [8, 9]. Conversely, urine can be readily collected from the vast majority of patients, and we [7] and others [10] have previously demonstrated that Xpert MTB/RIF testing of urine samples concentrated by centrifugation assay provides a substantial diagnostic yield of TB in this patient population. However, implementing such a strategy in resource-limited settings can be hindered by the cost, and the need for laboratory infrastructure with a reliable electricity supply to allow urine centrifugation and Xpert MTB/RIF testing. In the present study, we therefore explored the utility of the Determine TB-LAM^©^ (Alere Inc., Waltham, MA, USA) lateral-flow urine assay—a technically much simpler, instrumentation-free, low-cost, point-of-care diagnostic tool [11]. We reasoned that this could be used to test urine samples within the emergency admission unit or ward environment without the need for laboratory infrastructure, and might therefore be more feasibly implemented at more remote health facilities in resource-limited settings. In this study, we report on the diagnostic accuracy of the Determine TB-LAM test and its incremental diagnostic yield when used in combination with sputum-based diagnosis. We also report on the strong association between urine-lipoarabinomannan (LAM) status and mortality during 90 days of follow-up.

## Methods

### Setting and patients

This study forms part of a larger study of rapid urine-based approaches to diagnosis of HIV-associated TB that has previously been reported [7]. The prospective, observational cohort reported herein was enrolled at G.F. Jooste Hospital in Cape Town, South Africa. This 200-bed adult district hospital serves township communities in which there is a high seroprevalence of HIV and incidence of TB. This study was approved by the Human Research Ethics Committees of the University of Cape Town and the London School of Hygiene & Tropical Medicine. Patients provided written informed consent in their first language. The study is reported according to STARD initiative guidelines for reporting studies of diagnostic accuracy [12].

Adult patients aged ≥18 years were recruited on 4 days of the week from medical wards. On recruitment days, the study nurse coordinator ascertained from the ward register all medical admissions in the previous 24-hour period and recorded these in the study register. All patients who either previously tested negative or who had undocumented HIV status were offered HIV testing using two approved rapid tests. All patients who had a current TB diagnosis and/or were receiving treatment for TB at the time of hospital admission were excluded from the present study. Otherwise, all other adult patients with documented positive HIV status were eligible for the study and were invited to participate regardless of presenting symptoms or reason for hospital admission.

### Procedures and samples

Demographic and clinical details (including the World Health Organization [WHO] four-symptom screen for HIV-associated TB [13]) were recorded. The research team systematically investigated patients by obtaining sputum, urine and blood specimens for TB investigations between 9.00 a.m. on the day of enrolment and 9.00 a.m. the following day. Numerous additional samples (including both sputum and non-respiratory samples) for mycobacteriology were obtained by the routine medical team according to clinical indication throughout the admission period (Additional file 1: Table S1).

In the initial 24-hour period of hospital admission, two sputum samples were requested from each patient with careful instruction and supervision by the study nurse coordinator (an experienced respiratory nurse) as previously described [7]. A spot specimen was obtained first, followed by a second sample, which was induced using nebulized 3% hypertonic saline. When necessary, both specimens were induced. Alternatively, for those either too unwell to leave the ward to attend the sputum induction facility or too unwell for the sputum induction procedure, two spot specimens were requested. Urine samples were systematically collected in single-use disposable bedpans (Litha Healthcare Group, Johannesburg, South Africa). Fresh aliquots (2.0 mL) were sent for Xpert testing and the remaining urine volume (30–40 mL) was stored at –20 °C. Venous blood (5.0 mL) was inoculated into BACTEC™ Myco F/Lytic culture vials (Becton Dickinson, Franklin Lakes, NJ, USA).

### Laboratory procedures

Specimens were processed using standardized protocols in centralized accredited laboratories of the South African National Health Laboratory Service as described previously [14, 15]. Decontaminated, centrifuged deposits of sputum samples were re-suspended in phosphate buffer. Smears stained with auramine O stain were examined with fluorescence microscopy. Equal volumes were tested using Xpert and culture in mycobacterial growth indicator tubes (MGIT; Becton Dickinson, Sparks, MD, USA), which were incubated for up to 6 weeks. Culture isolates were identified as *M. tuberculosis* complex with the MTBDR*plus* assay (Hain Lifesciences, Nehren, Germany). Non-respiratory samples were also tested using MGIT liquid culture, with the exception of venous blood, which was tested using Myco F/Lytic culture.

Urine was tested using Xpert MTB/RIF in two different ways as described previously [7]. Fresh urine samples (2.0 ml) were centrifuged and the pellet re-suspended in 0.75 mL of phosphate buffer before testing using the Xpert MTB/RIF assay [7]. Each stored urine sample of between 30 and 40 mL was thawed and centrifuged at 3000 × *g* for 15 min. Following removal of the supernatant, the pellet was re-suspended in the residual urine volume and 0.75 mL was tested using Xpert. This second sample derived from centrifugation of a large urine volume was referred to as the ‘concentrated’ urine sample and these samples were batch processed on a weekly basis due to study-related logistical considerations and laboratory workflow.

The same frozen urine samples (unconcentrated and unprocessed) were also retrospectively tested for the presence of LAM using the Determine TB-LAM test (Alere Inc.) following the manufacturer’s instructions. Samples were thawed to ambient temperature and for each sample, 60 μL of unprocessed urine was applied to the sample pad at the bottom of the test strip. After 25 min, test strips were independently read by two investigators (SDL and MV) blinded to patient status and all other test results. Having checked for the presence of the positive control band on each strip, test results were scored as grade 1, 2, 3, 4 or 5 when compared to the reference card as described elsewhere [13]. For example, for the test result to be scored as grade 2, the test band had to be at least as intense as the corresponding grade 2 band on the reference card but not as intense as the grade 3 reference band. Any discrepancies in scoring were reviewed by both readers and the result resolved and agreed within 35 min of initial urine application. After resolution of results, the grade scores were categorized as being ‘positive’ (grade 2 or higher) or ‘negative’ (no test band at least as intense as grade 2).

Results of all microbiological tests were returned to the clinical team to inform treatment decisions with the exception of the results of the Determine TB-LAM assay, which was not endorsed for clinical use in South Africa or by the WHO at the time of the study.

### Clinical outcomes

Patient and ward records as well as district, regional and national electronic data systems were used to ascertain deaths during the first 90 days following enrolment as fully described elsewhere [16]. Patients with an unconfirmed vital status at 90 days (<5%) were considered to be lost to follow-up and data were censored on the last date of contact with the health system.

### Data analysis

#### Diagnostic yield of Determine TB-LAM and relationship with patient symptoms and other characteristics

The diagnostic reference standard for a confirmed new TB diagnosis was the detection of *M. tuberculosis* from at least one clinical sample of any type using MGIT culture and/or Xpert MTB/RIF. The total number of confirmed cases (*n* = 139) was used as the denominator to calculate the comparative diagnostic yield obtained from different sample types and different assays, including Determine TB-LAM. The yield of TB diagnoses from Determine TB-LAM testing of urine samples obtained during the initial 24 hours was compared with the yield from sputum samples collected in the same period. We included Xpert results in our diagnostic reference standard. While we calculated the yield of diagnoses made by Xpert in different specimen types compared with other methods, this does not represent a measure of diagnostic sensitivity (calculation of sensitivity was not performed). Diagnostic yield in our study reflected both the performance of the diagnostic test and the ability to obtain samples for that test in a real-world clinical setting. The associations between the yield of Determine TB-LAM and patient symptoms and other patient characteristics were explored and multivariable logistic regression was used to identify factors independently associated with positive urine-LAM status among patients with confirmed TB. One or more positive reference standard tests on a non-respiratory sample was taken as evidence of extrapulmonary TB (EPTB).

#### Diagnostic accuracy of Determine TB-LAM

For the purposes of assessing the diagnostic accuracy (sensitivity and specificity) of Determine TB-LAM, we identified a cohort of patients for whom sufficient microbiological data and the results of Determine TB-LAM urine testing were available. For inclusion in this analysis, patients were required to have the results of reference standard tests (liquid culture and/or Xpert MTB/RIF) from two or more samples obtained from two or more different anatomic sites, such as blood, sputum, urine or other non-respiratory samples. The rationale for this was to reduce the risk of patients with disseminated TB having false-negative reference standard tests and being inappropriately designated as ‘TB-free’. By doing so, we enhanced the methodological rigour with which the specificity of Determine TB-LAM was assessed [17]. From this cohort, those testing positive for *M. tuberculosis* were used as the denominator to calculate the sensitivity of Determine TB-LAM (*n* = 136). In contrast, patients whose clinical samples all tested negative for *M. tuberculosis* were defined as ‘TB-free’ and were used as the denominator to calculate the specificity of Determine TB-LAM (*n* = 277).

#### Patient characterization

Patients were characterized using simple descriptive statistics. Anaemia severity was defined using WHO criteria: no anaemia (haemoglobin concentration ≥13.0 g/dL for males, ≥12.0 g/dL for females), mild anaemia (11.0–12.9 g/dL for males, 11.0–11.9 g/dL for females), moderate anaemia (8.0–10.9 g/dL for males and females) or severe anaemia (<8.0 g/dL for males and females) [18]. TB prevalence with 95% exact confidence intervals (95% CI) was calculated. Medians were compared using either Wilcoxon rank-sum tests or Kruskal–Wallis tests as appropriate and means were compared using unpaired *t* tests. Chi-squared, Fisher’s exact and McNemar’s tests were used as appropriate to compare proportions. Kaplan–Meier plots were used to examine mortality risk among confirmed TB cases stratified by urine-LAM status and a Cox proportional hazards model was used to determine factors associated with mortality. Person-time was accrued from the date of study enrolment until death, loss to follow-up or censorship 90 days after study entry. All variables in the univariable model meeting a cut-off of *P* ≤ 0.1 were included in the multivariable model. Statistical tests were two-sided at α = 0.05.

## Results

### Patients enrolled and their characteristics

**Fig. 1:**
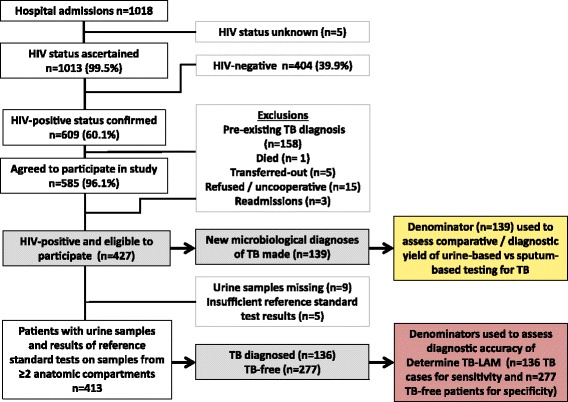
Flow diagram showing the study population and the numbers of patients included. *HIV* human immunodeficiency virus, *TB* tuberculosis

Between 6 June 2012 and 4 October 2013, the HIV status of 1013 of 1018 (99.5%) unselected new admissions to the adult medical wards was ascertained (Fig. 1). Of the 609 patients with a documented positive HIV test, 585 (96.1%) were willing to participate in the study. Those already receiving treatment for an existing diagnosis of TB at the time of admission (*n* = 158, 27.0%) were excluded, leaving 427 eligible patients who formed the study cohort (Fig. 1).

Patients were typically young adults; a majority knew their positive HIV status before admission and more than half of all patients had previously started antiretroviral therapy (ART) (Table 1). Immunodeficiency was typically advanced and overall median CD4 cell counts were lower among those who were ART-naive compared to those currently receiving ART (115 cells/μL versus 212 cells/μL; *P* < 0.001). Nearly half of all patients had previously received treatment for TB and two-thirds had moderate or severe anaemia (Table 1).

**Table 1:** Characteristics of all patients stratified according to tuberculosis status

	All patients (*n* = 427)	Patients with a TB diagnosis (*n* = 139)	Patients without a TB diagnosis (*n* = 288)	*P* value
Age (years), median (IQR)	36.1 (28.9–42.4)	33.8 (27.2–39.7)	37.1 (30.0–44.0)	0.008
Female	259 (60.7)	90 (64.8)	169 (58.7)	0.229
New HIV diagnosis on admission	82 (19.2)	34 (24.5)	48 (16.7)	0.055
TB characteristics				
History of previous TB*	196 (46.1)	50 (36.2)	146 (50.9)	0.005
RIF resistance	4 (0.9)	4 (2.9)	0	0.004
ART status				
ART-naive	177 (41.5)	71 (51.1)	106 (36.8)	0.019
ART interrupted	71 (16.6)	19 (13.7)	52 (18.1)	
Current ART use	179 (41.9)	49 (35.3)	130 (45.1)	
Treatment duration if currently on ART (years), median (IQR)^a^	1.6 (0.6–3.6)	1.3 (0.2–2.9)	1.7 (0.6–3.7)	0.157
CD4 counts (cells/μL^)^*				
Median (IQR)	149 (55–312)	80 (33–182)	191 (73–392)	<0.001
<50	94 (22.2)	53 (38.4)	41 (14.3)	
50–99	66 (15.6)	21 (15.2)	45 (15.7)	
100–149	54 (12.7)	19 (13.8)	35 (12.2)	
150–199	42 (9.9)	16 (11.6)	26 (9.1)	
≥200	169 (39.9)	29 (21.0)	140 (49.0)	
HIV viral load (log copies/mL)^b^				
Median (IQR)	4.4 (1.6–5.5)	5.1 (3.3–5.8)	4.0 (1.6–5.4)	<0.001
Virally suppressed (<400 copies/mL)	131 (31.6)	25 (19.8)	106 (36.8)	0.001
Haemoglobin levels*				
Median (IQR)	9.6 (7.7–11.6)	8.4 (6.8–10.2)	10.2 (8.2–12.2)	<0.001
No/mild anaemia	144 (34.2)	28 (20.1)	116 (41.1)	
Moderate/severe anaemia	277 (65.8)	111 (79.9)	166 (58.9)	
C-reactive protein (mg/L)^c^				
Median (IQR)	73 (17–153)	112 (70–182)	54 (12–135)	<0.001
>50	246 (61.2)	108 (82.4)	138 (50.9)	<0.001
Symptoms				
Cough ≥2 weeks*	34 (8.0)	18 (13.0)	16 (5.6)	0.008
Sputum production*	161 (38.3)	51 (37.5)	110 (38.7)	0.808
Current cough*	201 (47.4)	84 (60.9)	117 (40.9)	<0.001
Current fever*	63 (14.9)	24 (17.5)	39 (13.6)	0.294
Current night sweats*	174 (41.0)	73 (52.9)	101 (35.3)	0.001
Current reported weight loss*	189 (44.5)	76 (55.1)	113 (39.4)	0.002
Positive WHO symptoms screen*	389 (91.5)	134 (97.1)	255 (88.9)	0.005

Data are presented as n (%), unless otherwise stated. *Between 2 and 7 values missing, ^a^limited to among 179 patients currently receiving ART, ^b^13 results missing, ^c^20 results missing.

*Abbreviations*: *ART* antiretroviral therapy, *IQR* interquartile range, *RIF* rifampicin, *TB* tuberculosis, *WHO* World Health Organization

### Samples tested and laboratory-confirmed TB diagnoses made

During hospital admission, clinical samples for mycobacteriology were obtained from all 427 eligible study participants. The total of 1745 samples (mean 4.1 samples per patient) were derived from a median of three anatomic locations (Additional file 1: Table S1). These yielded 2391 reference standard test results (mycobacterial liquid culture and Xpert MTB/RIF). Results were missing for 4.3% of sputum cultures (*n* = 12), which were contaminated, and 4.3% of sputum Xpert tests (*n* = 12) and 3.1% of urine Xpert tests (*n* = 26), which had indeterminate results. However, all urine samples yielded interpretable LAM results (*n* = 418). TB was diagnosed in 139 patients with a mean of 3.2 positive samples per case diagnosed. Thus, the overall TB prevalence was 32.6% (95% CI 28.1–37.2). The mean number of tests done was similar for patients in whom TB was or was not diagnosed (5.8 tests versus 5.5 tests, respectively; *P* = 0.154, indicating a comparable intensity of investigation). The characteristics of those with and without TB are summarized in Table 1. Compared with those who were TB-free, those with a new TB diagnosis had lower CD4 counts and lower haemoglobin levels. The vast majority of patients had a positive WHO symptom screen regardless of TB status, but only one-third of patients (with or without TB) reported sputum production prior to admission.

**Table 2:** Diagnostic yield of tuberculosis cases from different sample types and the proportion of each diagnosed by Determine TB-LAM

			Proportion diagnosed by Determine TB-LAM (true positives)	
Samples from which TB diagnoses were made	Number of TB diagnoses	Diagnostic yield, % (95% CI)	Number	Proportion, % (95% CI)
All clinical samples and tests	139	100	53	38.1 (30.0–46.7)
Study samples collected during the first 24 h of admission				
Urine				
Urine Xpert (unconcentrated)	59	42.4 (34.1–51.1)	37	62.7 (49.1–75.0)
Urine Xpert (concentrated)	82	59.0 (50.3–67.3)	43	52.4 (41.1–63.6)
Sputum				
Smear microscopy	27	19.4 (13.2–27.0)	15	55.6 (35.3–74.5)
Xpert (1st sample only)	37	26.6 (19.5–34.8)	17	45.9 (29.5–63.1)
Xpert (either sample)	39	28.1 (20.8–36.3)	17	43.6 (27.8–60.4)
All study samples collected during any point during admission				
All EPTB cases*	113	81.3 (73.8–87.4)	51	45.1 (35.8–54.8)
Blood	41	29.5 (22.1–37.8)	27	65.9 (49.4–79.9)
Pleural fluid	13	9.4 (5.1–15.5)	4	30.8 (9.0–61.4)
Urine^a^	92	66.2 (57.9–74.0)	46	50.0 (39.4–60.6)
Cerebrospinal fluid	8	5.8 (2.5–11.0)	1	12.5 (0.3–52.7)
Lymph node fine needle aspirate	6	4.3 (1.6–9.2)	4	66.7 (22.3–95.7)

*All patients who had evidence of extrapulmonary involvement, i.e. ≥1 non-respiratory sample of any type that tested positive by culture and/or Xpert (regardless of other results on respiratory samples); ^a^culture and/or Xpert testing.

*Abbreviations*: *CI* confidence interval, *EPTB* extrapulmonary tuberculosis, *TB* tuberculosis

**Table 3:** Characteristics of patients with tuberculosis stratified according to Determine TB-LAM assay result

	Among TB patients by LAM status (n = 139)			
	LAM-positive (*n* = 53)	LAM-negative (*n* = 83)	No LAM result (*n* = 3)	*P* value*
Age (years), median (IQR)	33.4 (25.9–40.6)	34.2 (28.2–38.7)	25.3 (25.1–32.4)	0.582
Female	30 (56.6)	57 (68.7)	3 (100)	0.153
New HIV diagnosis on admission	19 (35.9)	14 (16.9)	1 (33.3)	0.012
History of previous TB^a^	17 (32.1)	31 (37.8)	2 (66.7)	0.497
ART status				
ART-naive	28 (52.8)	42 (50.6)	1 (33.3)	0.058
ART interrupted	11 (20.8)	7 (8.4)	1 (33.3)	
Current ART use	14 (26.4)	34 (41.0)	1 (33.3)	
If currently on ART, treatment duration (years), median (IQR)*	0.1 (0–2.5)	1.5 (0.6–4.1)	0.8 (0.8–0.8)	0.055
CD4 counts (cells/uL)^a^				
Median (IQR) cells/μL	42 (23–91)	140 (47–247)	74 (22–546)	<0.001
<50	30 (56.6)	22 (26.8)	1 (33.3)	<0.001
50-99	11 (20.8)	9 (11.0)	1 (33.3)	
100-149	7 (13.2)	12 (14.6)	0	
150-199	4 (7.6)	12 (14.6)	0	
≥200	1 (1.9)	27 (32.9)	1 (33.3)	
HIV viral load (log copies/mL)^b^				
Median (IQR)	5.4 (4.3–6.0)	4.7 (2.5–5.5)	4.5 (3.2–5.9)	0.005
Virally suppressed (<400 copies/mL)	4 (8.0)	21 (26.6)	0	0.011
Anaemia category				
Median (IQR)	7.6 (6.5–8.6)	9.0 (7.4–11.1)	7.1 (6.9–11.8)	<0.001
No/mild anaemia	3 (5.7)	24 (28.9)	1 (33.3)	0.001
Moderate/severe anaemia	50 (94.3)	59 (71.1)	2 (66.7)	
C-reactive protein (mg/L)^b^				
Median, (IQR)	168 (107–204)	95 (44–142)	100 (87–134)	<0.001
<50	0	23 (29.5)	0	<0.001
≥50	50 (100)	55 (70.5)	3 (100)	
Symptoms				
Cough ≥2 weeks^a^	8 (13.2)	11 (13.4)	0	0.972
Sputum production^a^	22 (41.5)	27 (33.8)	2 (66.7)	0.364
Current cough^a^	31 (58.5)	22 (62.2)	2 (66.7)	0.667
Current fever^a^	22 (41.5)	27 (33.8)	2 (66.7)	0.436
Current night sweats	11 (21.2)	13 (15.9)	0	0.965
Current reported weight loss^a^	30 (56.6)	44 (53.7)	2 (66.7)	0.737
Positive WHO symptoms screen^a^	52 (98.1)	79 (96.3)	3 (100)	0.553
RIF resistance				
Yes	1 (1.9)	3 (3.6)	0	1

Data are presented as n (%), unless otherwise stated. *Limited to among 49 patients currently receiving ART, ^a^between 1 and 3 values missing, ^b^8 values missing. *Abbreviations*: *ART* antiretroviral therapy, *IQR* interquartile range, *LAM* lipoarabinomannan, *WHO* World Health Organization

### Diagnostic accuracy of Determine TB-LAM urine testing

Having carefully defined which patients in the cohort had TB and which were TB-free, the diagnostic accuracy of urine-LAM was next assessed using data from the subset of 413 patients (96.7%) for whom reference standard test results on samples obtained from at least two anatomic sites plus urine-LAM results were available and complete (Fig. 1). In this subset of patients, TB was diagnosed in 136 patients and this was used as the denominator to calculate the sensitivity of Determine TB-LAM. Culture and Xpert tests were negative on all samples obtained from the remaining patients (*n* = 277), who were therefore designated as ‘TB-free’ and used to assess the specificity of Determine TB-LAM. Among these 277 patients, the mean number of negative reference standard tests per patient was 5.5.

Using the grade 2 reference band as the cut-off to designate urine-LAM results as ‘positive’ or ‘negative’ [13], the Determine TB-LAM results of the two independent readers of the lateral-flow tests were concordant in 407 of 413 tests, giving a proportionate agreement of 98.5% (95% CI 96.7–99.5) and a kappa statistic of 0.937 (95% CI 0.887–0.987). Following review of the discordant results from the remaining six tests, the two readers readily agreed upon a consensus result for each. Thus, TB-LAM results in agreement were available for all 413 patients.

Urine-LAM was positive in 53 of 136 TB cases, giving an overall sensitivity of 39.0% (95% CI 30.7–47.7). Urine-LAM was negative in 274 of the 277 patients who were TB-free, giving a specificity of 98.9% (95% CI 96.9–99.8). Two of three patients with apparent false-positive urine-LAM results had clinico-radiological diagnoses of TB made by the routine medical team, although diagnoses were never confirmed microbiologically. Of note, neither had a full set of investigations available, with sputum and blood culture results missing. One patient had a 3-week history of weakness, diarrhoea and constitutional symptoms, was pancytopaenic (grade 2 LAM-positive), and subsequently died without any diagnosis being made. The other patient had a 3-month history of abdominal pain and weight loss, and an abdominal ultrasound demonstrated splenomegaly and splenic micro-abscesses with para-aortic and mesenteric lymphadenopathy suggestive of abdominal TB (grade 5 LAM-positive). Thus, had a composite reference standard for TB diagnosis (all microbiological and clinico-radiological diagnoses) been used in a post hoc analysis, the specificity would have been 279 out of 280 TB-free patients (99.6%; 95% CI 98.0–100). Using a grade 1 reference band cut-off resulted in modest improvement in sensitivity with a small decrease in specificity (Additional file 2: Table S2). Both grade 1 and 2 reference band cut-offs had improved sensitivity when restricted to those with CD4 counts <100 cells/μL (56.9% and 68.1% using grade 2 and grade 1 cut-offs, respectively).

### Comparative diagnostic yield of urine and sputum samples obtained in the first 24 hours

Given the very high specificity of urine-LAM, a positive Determine TB-LAM result was indicative of TB (positive predictive value [95% CI 85.1-98.9]). We therefore next compared the diagnostic yield of urine-LAM, sputum-Xpert and sputum microscopy from samples obtained in the first 24 hours of admission from all 427 included patients.

The potential yield of sputum-based diagnoses was substantially limited by the high proportion of patients who were unable to produce a sputum sample. In the first 24 hours of admission only 158 of 427 (37.0%) patients were able to produce at least one sputum sample (spot and/or induced samples) despite assistance from an experienced respiratory nurse. Of these sputum samples, 36 (23.1%) were only obtained following sputum induction. The remaining patients who did not produce sputum were typically too sick or uncooperative to tolerate sputum induction or to be taken to the sputum induction room at the hospital, or it was attempted but was simply unsuccessful. In marked contrast, urine samples were readily obtained from 425 of 427 (99.5%) patients, although seven samples were misplaced in transit to the laboratory, leaving 418 urine samples available for testing (Fig. 1).

Sputum samples were eventually produced by a total of 245 patients (57.4%) at some point during their hospital admission. Of the 161 patients who self-reported sputum production prior to admission, 143 (88.8%) were able to produce a sputum sample for testing. Additionally, of the 201 patients reporting current cough at admission, 159 (79.1%) produced a sputum sample.

**Fig. 2:**
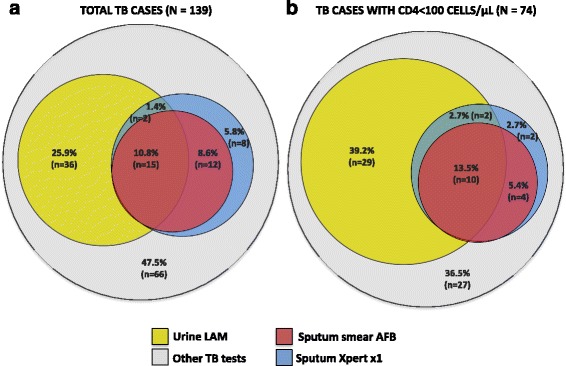
Venn diagrams showing the proportions of (**a**) total tuberculosis (*TB*) diagnoses (*n* = 139) or (**b**) TB diagnoses in patients with CD4 cell counts <100 cells/μL (*n* = 74) and the proportions (diagnostic yields) which could be made using smear microscopy, sputum Xpert or urine-lipoarabinomannan (*LAM*) (these tests were all conducted on samples obtained within 24 hours of admission). *AFB* Acid-fast bacilli

Figure 2a shows the comparative diagnostic yield of urine-LAM and sputum-based testing with microscopy and with Xpert MTB/RIF from samples taken in the first 24 hours. The diagnostic yields obtained from sputum microscopy, sputum-Xpert and urine-LAM were 19.4% (*n* = 27/139), 26.6% (*n* = 37/139) and 38.1% (*n* = 53/139), respectively (comparison of the proportionate diagnostic yield from sputum microscopy with the yield from sputum-Xpert and urine-LAM: McNemar’s *P* < 0.01 for each comparison).

The incremental diagnostic yield of Determine TB-LAM used in combination with sputum testing is shown in Fig. 2a. The addition of urine-LAM testing to sputum microscopy increased the overall diagnostic yield from 19.4% to 46.8% (*n* = 65/139: a 2.4-fold increase; *P* < 0.001) of all TB cases. The addition of urine-LAM testing to sputum-Xpert increased the diagnostic yield from 26.6% to 52.5% (*n* = 73/139: a 2.0-fold increase; *P* < 0.001).

### Sub-analysis of patients with CD4 cell counts <100 cells/μL

Figure 2b shows the diagnostic yields obtained from sputum microscopy, sputum-Xpert and urine-LAM among those patients with CD4 cell counts <100 cells/μL (*n* = 74) for specimens obtained in the first 24 hours of admission. The diagnostic yield from sputum microscopy, sputum-Xpert and urine-LAM were 18.9% (*n* = 14/74), 24.3% (*n* = 18/74) and 55.4% (*n* = 41/74), respectively (comparison of the proportionate diagnostic yield from sputum microscopy with the yield from sputum-Xpert and urine-LAM: McNemar’s *P* = 0.13 and *P* < 0.001 for each comparison, respectively). The addition of urine-LAM testing to sputum microscopy increased the diagnostic yield from 18.9% to 60.8% (*n* = 45/74: a 3.2-fold increase; *P* < 0.001). The addition of urine-LAM testing to sputum-Xpert increased the diagnostic yield from 24.3% to 63.5% (*n* = 47/74; 2.6-fold increase; *P* < 0.001). Thus, the incremental diagnostic yield of urine-LAM was greater among patients with lower CD4 counts (Fig. 2a, b).

### Relationship between patient characteristics and respiratory symptoms and the diagnostic yield of Determine TB-LAM

**Fig. 3:**
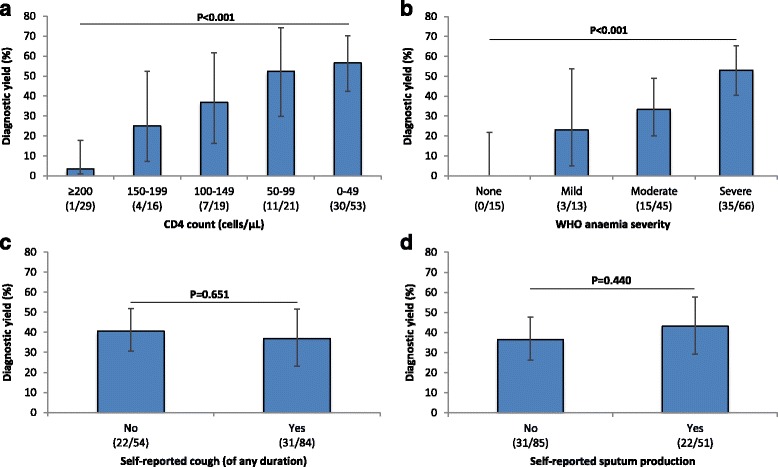
Bar charts displaying the diagnostic yields of urine-lipoarabinomannan (LAM) testing stratified according to (**a**) patient CD4 cell count, (**b**) World Health Organization anaemia severity categorization, (**c**) self-reported cough and (**d**) self-reported sputum production. The numbers of patients in each stratum that were urine-LAM-positive are shown beneath each chart

The diagnostic yield of Determine TB-LAM was substantially greater among patients with lower CD4 cell counts, with a maximum yield of 56.6% (95% CI 45.4-67.2) observed among those with CD4 counts of <50 cells/μL (Fig. 3a). Yield was also much higher among those with more severe anaemia, with the diagnostic yield rising to 53.0% (95% CI 43.0-62.8) among those classified as having ‘severe’ anaemia (Fig. 3b). In contrast, the yield did not vary according to whether patients complained of cough or sputum production (Fig. 3c, d). Of the 53 patients with confirmed TB who tested urine LAM-positive, 22 (41.5%) reported no cough at the time of admission and 31 (58.5%) had no history of sputum production during their illness. Thus, it was notable that urine-LAM identified an important subgroup of TB cases who had no respiratory symptoms. Moreover, 36 of 53 urine-LAM-positive cases (67.9%) were patients who were unable to produce a sputum sample within the first 24 hours of admission.

### Diagnostic yield among patients with pulmonary and extrapulmonary forms of HIV-associated tuberculosis

Of the patients whose TB was diagnosed using Xpert MTB/RIF testing of sputum obtained in the first 24 hours, 43.6% (*n* = 17) could be diagnosed by urine TB-LAM instead (Table 2). Overall, 113 patients (81.3%) had EPTB and of these 51 (45.1%) tested positive for urine-LAM, although the yield varied substantially by the type of EPTB. The proportion testing positive by TB-LAM was highest among those with positive blood cultures (65.9%) and those with positive lymph node aspirates (66.7%) (Table 2). The yield of TB-LAM tended to be lower among those with positive cultures of cerebrospinal fluid (12.8%) and pleural fluid (30.8%) (Table 2).

### Comparative diagnostic yield of urine-Xpert and Determine TB-LAM

The greatest yield of any diagnostic approach in this study was the Xpert testing of urine that had been concentrated by centrifugation. A single Xpert test on concentrated urine yielded 59.0% of total diagnoses (*n* = 82), compared with 39.1% (*n* = 53) for urine-LAM (Table 2). Of the patients with positive urine Xpert tests on a concentrated sample, 43 (52.4%) tested positive with the Determine TB-LAM assay. Additionally, 9 of 53 patients (16.9%) testing positive on urine-LAM had a negative urine Xpert result (using a concentrated sample).

### Comparison of the characteristics and mortality risk of tuberculosis patients with positive (*n* = 53) or negative (*n* = 83) urine-lipoarabinomannan status

Comparing the clinical phenotype of patients with confirmed TB stratified according to urine-LAM status revealed that those testing LAM-positive had lower CD4 cell counts and a higher prevalence and severity of anaemia (Table 3). In contrast, symptoms (respiratory or otherwise) were not associated with urine-LAM status. In multivariable analysis (Table 4), the only independent predictors of a positive urine-LAM status among confirmed TB patients (*n* = 136) were lower CD4 cell counts and more severe anaemia.

**Table 4:** Association between characteristics of patients with tuberculosis (*n* = 136) and detection of lipoarabinomannan in urine

	Unadjusted odds ratio (95% CI)	Log-rank	Adjusted odds ratio (95% CI)	Log-rank
Age (for each year decrease)	1.01 (0.98–1.05)	0.491		
Gender				
Female	1.00	0.154		
Male	1.68 (0.82–3.43)			
ART status				
Any ART exposure	1.00	0.748		
ART naive	1.12 (0.56–2.24)			
History of previous TB				
Yes	1.0	0.496		
No	1.29 (0.62–2.67)			
CD4 category (cells/uL)				
≥150	1.00	<0.001	1.00	0.002
100–149	4.55 (1.22–16.98)		4.37 (1.12–16.98)	
50–99	9.53 (2.64–34.35)		6.76 (1.78–25.65)	
<50	10.64 (3.61–31.36)		7.20 (2.34–22.17)	
Anaemia category				
None/mild	1.00	<0.001	1.00	0.051
Moderate	4.00 (1.03–15.44)		2.41 (0.57–10.24)	
Severe	9.66 (2.64–35.33)		4.62 (1.14–18.74)	
Self-reported sputum production				
No	1.00	0.264		
Yes	1.51 (0.73–3.13)			
Sputum produced in the first 24 hours				
Yes	1.00	0.841		
No	1.08 (0.52–2.25)			
Sputum sample produced during study admission				
Yes	1.00	0.749		
No	1.13 (0.54–2.35)			
WHO screen positive				
No	1.00	0.541		
Yes	1.97 (0.20–19.50)			

*Abbreviations*: *ART* antiretroviral therapy, *CI* confidence interval, *TB* tuberculosis, *WHO* World Health Organization

**Table 5:** Risk factors for mortality within 90 days of study entry among patients with confirmed tuberculosis (*n* = 139)

	Unadjusted hazard ratio (95% CI)	Log-rank	Adjusted hazard ratio (95% CI)	Log-rank
Age (for each year decrease)	1.01 (0.97–1.06)	0.545		
Gender				
Female	1.00	0.093	1.00	0.205
Male	2.17 (0.88–5.35)		1.80 (0.73–4.48)	
ART status				
Current ART	1.00	0.922		
ART naive	1.18 (0.43–3.24)			
Interrupted ART	1.29 (0.32–5.18)			
CD4 category (cells/uL)				
≥150	1.00	0.261		
199–149	3.30 (0.74–14.75)			
50–99	1.58 (0.26–9.48)			
<50	2.92 (0.80–10.61)			
HIV viral load (log copies/mL)				
<400	1.00	0.683		
≥400	1.30 (0.37–4.48)			
C-reactive protein (mg/L)				
<50	1.00	0.262		
≥50	3.16 (0.42–23.47)			
LAM-positive				
No	1.00	0.007	1.00	0.004
Yes	3.56 (1.35–9.36)		4.20 (1.50–11.75)	
RIF-resistant TB				
No	1.00	0.082	1.00	0.032
Yes	4.90 (1.13–21.24)		8.41 (1.74–40.67)	

*Abbreviations*: *ART* antiretroviral therapy; *CI* confidence interval, *LAM* lipoarabinomannan, *RIF* rifampicin, *TB* tuberculosis, *WHO* World Health Organization

**Fig. 4:**
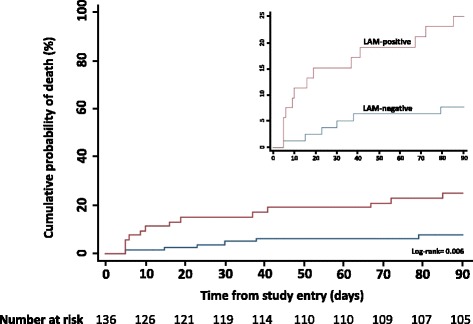
Cumulative probability of death within 90 days of study enrolment of patients with confirmed HIV-associated tuberculosis (*n* = 136 with urine samples available), stratified according to urine-lipoarabinomannan (*LAM*) status. For ease of viewing, a magnified view is shown in the *upper right*

Having demonstrated that a positive urine-LAM status was associated with the severity of illness, we next compared survival among patients who tested urine LAM-positive or LAM-negative (Fig. 4). Among the 136 TB patients with urine-LAM results available, 13 of 53 testing LAM-positive died (24.5%; 95% CI 13.8–38.3) compared with 6 of 83 testing LAM-negative (7.2%; 95% CI 2.7–15.1; log-rank *P* = 0.006). Therefore, urine-LAM detected TB in 13 of 19 patients (68.4%) with HIV-associated TB who died within 90 days of study entry. In unadjusted analyses, a greater mortality risk was also associated with male sex and rifampicin-resistant TB but not with CD4 cell count (Table 5). In adjusted analyses, mortality remained strongly associated with urine-LAM status (adjusted hazards ratio 4.20; 95% CI 1.50–11.75) and with rifampicin-resistant disease (Table 5).

## Discussion

As reported in the parent study, a microbiological diagnosis of TB was made in one in three unselected HIV-positive adult patients admitted to medical wards in this South African district hospital. The prevalence is so high in this vulnerable patient group that a simple, rapid screening test for TB that can be immediately performed at the time of hospital admission is highly desirable. In the present study, we found that routine urine-LAM testing could detect 39% of all HIV-associated TB diagnoses and the large majority of TB diagnoses among patients dying within 90 days of admission to hospital. We previously showed the remarkable utility of using Xpert MTB/RIF to test urine samples concentrated by centrifugation, with 64% of total diagnoses being made using samples obtained within the first 24 hours of admission (1.6-fold more diagnoses than LAM and 2.3-fold more than sputum Xpert testing). We are currently conducting a clinical trial in sub-Saharan Africa to assess whether implementation of systematic testing of urine samples using rapid TB assays compared to the current sputum-based strategy results in improved survival among HIV-patients requiring acute medical hospitlization (Rapid Urine-Based *S*creening for *T*uberculosis to Reduce *A*IDS-Related *M*ortality in Hospital In-Patients in Africa [STAMP] Trial; ISRCTN71603869) [19].

Should implementation of such a screening strategy (routine urine Xpert testing) be attempted in the countries of southern Africa worst hit by the HIV and TB epidemics, several potential barriers to the implementation would exist. These include the cost to purchase and maintain the GeneXpert platform, as well as the cost and supply chain of Xpert MTB/RIF cartridges (especially if cartridges are required to test both urine and sputum samples from each patient). Our screening strategy employing urine-based testing with Xpert MTB/RIF is also dependent upon the availability of laboratory facilities with sufficient capacity and biosafety equipment to undertake centrifugation of urine samples, including an uninterrupted electricity supply. All of these requirements may potentially undermine routine screening using Xpert testing of concentrated urine. It was for these reasons that we sought to explore the utility of the Determine TB-LAM lateral flow urine assay for LAM. The potential advantages of this diagnostic tool are self-evident: it currently costs approximately $2.66 per test (nearly one-quarter the cost of an Xpert MTB/RIF cartridge) and it requires no laboratory infrastructure, no laboratory personnel or expertise, and no instrumentation or electricity supply.

The main perceived limitation to the use of Determine TB-LAM is that it has only modest overall sensitivity [13, 20]. However, sensitivity is highest among the sickest patients with advanced immunodeficiency and worst prognostic characteristics, as seen in the present study and other previous observational studies [21]. Thus, the urine-LAM point-of-care test is ideal for use in HIV-positive inpatients requiring acute medical admission, as we enrolled in this study. Moreover, the limited sensitivity is greatly offset by the ease with which urine samples can be obtained from such patients compared to sputum. Whereas urine samples were readily obtained from almost all patients within the first 24 hours, just one in three patients could produce a sputum sample in the same time period despite the availability of sputum induction facilities. Thus, although the overall diagnostic yield from urine-LAM was modest (39%), it greatly exceeded the yield derived from sputum-based screening using fluorescence microscopy or Xpert MTB/RIF, and it doubled or more than doubled the diagnostic yield when used in addition to sputum-based screening.

Previous studies of the diagnostic accuracy of urine-LAM have restricted their evaluation to testing patients selected on the basis of their ability to produce sputum samples [8, 15, 22]. However, in the present study, just 51 (37.5%) of the 139 patients found to have a microbiological diagnosis of TB reported sputum production as a symptom during their illness, and only 37.0% could produce a sputum sample within the first 24 hours of admission. Of the true-positive urine-LAM results obtained, 31 of 53 (58.5%) were from patients who reported no sputum production (Fig. 3d). Thus, had urine-LAM screening been restricted to those with respiratory symptoms suggestive of pulmonary TB, almost three in five of the TB diagnoses that could be made using urine-LAM would have been missed. Among those with TB, we found no association between reported symptoms and a positive urine-LAM test in crude or adjusted analyses. Collectively, these findings indicate that urine-LAM screening would be most effective when done systematically on all HIV-infected patients sick enough to require hospital admission in high-TB-prevalence areas, without selection according to specific symptoms. Moreover, this suggests that previous studies that have selectively studied patients able to produce sputum are very likely to have under-estimated the true utility of urine-LAM screening [23].

Many researchers have previously suggested that urine-LAM assays lack sufficient specificity to be used as a ‘rule-in’ diagnostic test. Indeed studies have reported specificity to be as low as 75% [20]. In marked contrast, one of the most important findings of this study was that assay specificity was extremely high (98.9%), indicating that this assay has utility as a rule-in diagnostic test for TB and that the results could be used to confidently inform the decision to start TB treatment in this patient population. When reading the lateral-flow strips, we used the agreed consensus cut-off grade 2 band on the reference card [18]. The strips were easy to read with 98.5% agreement between the two independent, blinded readers. Previous studies have reported variable specificity (75–99%) [20] and this may not only relate to differences in interpretation of the test-strip bands, but also to the rigour of the microbiological reference standard [13]. We designed this study to optimize assessment of LAM specificity, with an average of 5.6 reference standard tests being done for each patient. Moreover, the clinical samples were obtained from a median of three different anatomic compartments. This very comprehensive microbiological reference standard likely underlies the high specificity we observed.

The diagnostic yield varied substantially with CD4 cell count, with no useful yield among those with counts >200 cells/μL, but with a gradually increasing yield within lower CD4 cell count strata, reaching a maximum of 56.6% among those with counts of 0–49 cells/μL. The yield from urine-LAM screening was independent of ART status. This study confirms that assay utility is restricted to HIV-infected patients with CD4 cell counts of <200 cells/μL as previously shown among HIV-infected medical inpatients [8, 22] and HIV-infected outpatients starting ART [15]. We also found a strong relationship between higher diagnostic yield and increasing severity of anaemia with a very poor overall yield among those with no or mild anaemia, as we have previously reported among outpatients [24]. Thus, patients with moderate or severe HIV-associated anaemia may especially benefit from TB screening using urine-LAM. In settings that do not permit universal TB screening due to limited resources, moderate or severe anaemia (in addition to advanced immunodeficiency) may serve as a simple marker for entry into the TB diagnostic algorithm.

During prospective follow-up, mortality was also found to be substantially greater among those testing urine-LAM positive in both crude and adjusted analyses. Other studies too have found a strong association between positive LAM status and mortality, including a recent meta-analysis that found a more than two-fold increased odds of mortality associated with LAM-positivity [21, 25–29]. Recently, a multi-country randomized controlled trial based in sub-Saharan Africa found that urine-LAM testing in addition to standard-of-care TB diagnostics was associated with a 17% (95% CI 4–28) relative risk reduction in 8-week all-cause mortality among HIV-infected patients requiring acute medical admission; in those with CD4 counts <50 cells/μL, the implementation of LAM was associated with a 29% (95% CI 10–44) relative risk reduction in 8-week all-cause mortality [30]. This was the first randomized controlled trial to demonstrate a mortality reduction associated with the implementation of a TB detection assay [31].

The Determine TB-LAM assay underwent expert review by the WHO in 2015. On the basis of a Cochrane review of all available data (that included unpublished data from the present study) [20] as well as the randomized trial by Peter et al. demonstrating a mortality benefit associated with LAM [30], the WHO now conditionally recommends that urine-LAM testing be used for all HIV-positive patients with CD4 counts <100 cells/μL who require hospitalization and have signs and symptoms of TB, as well as for HIV-positive inpatients who are seriously ill, irrespective of their CD4 cell count. While the sensitivity of the assay is generally modest, various factors favour implementation of this assay as a rule-in test for TB among HIV-positive inpatients by national TB programmes in resource-limited settings [32]. These include a proven mortality benefit, extremely high specificity and positive predictive value when a grade 2 cut-off is used, low-cost (consumable cost of $2.66 per test strip), and that it can be done at the point of care without laboratory expertise or instrumentation [22, 33].

Strengths of this study include the recruitment of consecutive admissions without selection according to presenting symptoms or other criteria. Patients were very thoroughly investigated, yielding a large number of TB diagnoses and providing a comprehensive reference standard to assess the diagnostic accuracy of urine-LAM. We believe that this has provided the most rigorous assessment of the diagnostic accuracy of Determine TB-LAM to date. Patients were well characterized and are likely to be representative of HIV-infected medical admissions in countries with a high TB burden. The consistency in findings of post-mortem studies reporting on the burden of undiagnosed disseminated TB in HIV-infected inpatient deaths across the African continent [6] suggests that these findings may be of relevance throughout Africa. Outcome data at 90 days (~95% available) allowed the prognostic value of urine-LAM to be carefully assessed. Weaknesses include the fact that sputum could only be obtained from 57.4% of participants during their admission despite access to sputum induction facilities. This may have resulted in under-ascertainment of pulmonary TB cases. However, the large numbers of extrapulmonary specimens obtained (Additional file 1: Table S1) likely minimized the under-diagnosis of TB. The low proportion of patients who could provide a sputum sample despite the availability of sputum induction facilities was an important reason for the relatively poor yield of sputum diagnostics in this study. This in part reflects the design of the study, which involved screening all HIV-infected patients whether they reported a cough or not (approximately 80% of those who self-reported a cough were able to produce a sputum sample). Had the proportion producing sputum been higher (e.g. through enhanced sputum induction procedures and transporting sicker patients to the induction facility with medical support), then the proportion of participants diagnosed using urine-LAM alone (rather than sputum Xpert or smear microscopy) may have been lower. However, sputum induction facilities and resources for additional medical support in hospital services are for the most part not available in sub-Saharan Africa. Another weakness is that the study was done at a single site and urine-LAM testing was done retrospectively on frozen samples. However, the feasibility of prospectively testing urine samples at the point of care has been demonstrated in other studies [22, 30]. Our results cannot be generalized to the testing of HIV-infected children, in whom the assay has unfortunately not been found to have utility [34]. Ongoing research on pathogen and host diagnostic urine biomarkers for TB may yield urine assays with improved performance characteristics in the future [35–37].

## Conclusions

We have rigorously evaluated the Determine TB-LAM diagnostic urine assay and demonstrated that its specificity is extremely high and it provides substantial incremental diagnostic yield when used as a routine screening test on unselected HIV-infected adults newly admitted to hospital. The addition of urine-LAM screening doubled the diagnostic yield derived from systematic screening of sputum using Xpert MTB/RIF. Much of the benefit from urine-LAM testing was among TB patients without respiratory symptoms or sputum production. In conjunction with recently published WHO recommendations, this study further supports the implementation of the Determine TB-LAM lateral-flow assay as an initial, routine point-of-care rule-in test for TB among HIV-patients requiring hospitalization in settings with a high TB burden.

## Additional files

Additional file 1: Table S1. Total clinical samples (‘research’ and ‘routine’ samples) sent for mycobacterial testing. (DOC 42 kb)
Additional file 2: Table S2. Diagnostic utility of urine-LAM by reference band cut-off and stratified by CD4 cell count. (DOCX 12 kb)

## References

[1] WHO. Global Tuberculosis Report 2016. Geneva: World Health Organization; 2016. http://apps.who.int/iris/bitstream/10665/250441/1/9789241565394-eng.pdf?ua=1. Accessed 26 Dec 2016

[2] UNAIDS (2015). World AIDS Day 2015 - Fact Sheet.

[3] Cohen T, Murray M, Wallengren K, Alvarez GG, Samuel EY, Wilson D (2010). The prevalence and drug sensitivity of tuberculosis among patients dying in hospital in KwaZulu-Natal, South Africa: a postmortem study. PLoS Med.

[4] Cox JA, Lukande RL, Nelson AM, Mayanja-Kizza H, Colebunders R, Van Marck E, Manabe YC (2012). An autopsy study describing causes of death and comparing clinico-pathological findings among hospitalized patients in Kampala, Uganda. PLoS One..

[5] Wong EB, Omar T, Setlhako GJ, Osih R, Feldman C, Murdoch DM, Martinson NA, Bangsberg DR, Venter WDF (2012). Causes of death on antiretroviral therapy: a post-mortem study from South Africa. PLoS One..

[6] Gupta RK, Lucas SB, Fielding KL, Lawn SD (2015). Prevalence of tuberculosis in post-mortem studies of HIV-infected adults and children in resource-limited settings: a systematic review and meta-analysis. AIDS..

[7] Lawn SD, Kerkhoff AD, Burton R, Schutz C, van Wyk G, Vogt M, Pahlana P, Nicol MP, Meintjes G (2015). Rapid microbiological screening for tuberculosis in HIV-positive patients on the first day of acute hospital admission by systematic testing of urine samples using Xpert MTB/RIF: a prospective cohort in South Africa. BMC Med..

[8] Peter JG, Theron G, van Zyl-Smit R, Haripersad A, Mottay L, Kraus S, Binder A, Meldau R, Hardy A, Dheda K (2012). Diagnostic accuracy of a urine lipoarabinomannan strip-test for TB detection in HIV-infected hospitalised patients. Eur Respir J..

[9] Reid MJA, Shah NS (2009). Approaches to tuberculosis screening and diagnosis in people with HIV in resource-limited settings. Lancet Infect Dis..

[10] Peter JG, Theron G, Muchinga TE, Govender U, Dheda K (2012). The diagnostic accuracy of urine-based Xpert MTB/RIF in HIV-infected hospitalized patients who are smear-negative or aputum scarce. PLoS One..

[11] Lawn SD (2012). Point-of-care detection of lipoarabinomannan (LAM) in urine for diagnosis of HIV-associated tuberculosis: a state of the art review. BMC Infect Dis..

[12] Bossuyt PM, Reitsma JB, Bruns DE, Gatsonis CA, Glasziou PP, Irwig LM, Lijmer JG, Moher D, Rennie D, de Vet HCW, Standards for Reporting of Diagnostic Accuracy (2003). Towards complete and accurate reporting of studies of diagnostic accuracy: The STARD Initiative. Ann Intern Med.

[13] Lawn SD, Dheda K, Kerkhoff AD, Peter JG, Dorman S, Boehme CC, Nicol MP (2013). Determine TB-LAM lateral flow urine antigen assay for HIV-associated tuberculosis: recommendations on the design and reporting of clinical studies. BMC Infect Dis..

[14] Lawn SD, Brooks SV, Kranzer K, Nicol MP, Whitelaw A, Vogt M, Bekker L-G, Wood R (2011). Screening for HIV-associated tuberculosis and rifampicin resistance before antiretroviral therapy using the Xpert MTB/RIF assay: a prospective study. PLoS Med..

[15] Lawn SD, Kerkhoff AD, Vogt M, Wood R (2012). Diagnostic accuracy of a low-cost, urine antigen, point-of-care screening assay for HIV-associated pulmonary tuberculosis before antiretroviral therapy: a descriptive study. Lancet Infect Dis..

[16] Meintjes G, Kerkhoff AD, Burton R, Burton R, Boulle A, van Wyk G, Blumenthal L, Nicol MP, Lawn SD (2015). HIV-related medical admissions to a South African district hospital remain frequent despite effective antiretroviral therapy scale-up. Medicine..

[17] Lawn SD, Kerkhoff AD, Nicol MP, Meintjes G (2015). Underestimation of the true specificity of the urine lipoarabinomannan point-of-care diagnostic assay for HIV-associated tuberculosis. J Acquir Immune Defic Syndr..

[18] World Health Organization. Haemoglobin concentrations for the diagnosis of anaemia and assessment of severity. Vitamin and mineral nutrition information system. Geneva: World Health Organization; 2011. http://www.who.int/vmnis/indicators/haemoglobin.pdf. Accessed 25 May 2016.

[19] Gupta-Wright A, Fielding KL, van Oosterhout JJ, Wilson DK, Corbett EL, Flach C, Reddy KP, Walensky RP, Peters JA, Alufandika-Moyo M, Lawn SD (2016). Rapid urine-based screening for tuberculosis to reduce AIDS-related mortality in hospitalized patients in Africa (the STAMP trial): study protocol for a randomised controlled trial. BMC Infect Dis..

[20] Shah M, Hanrahan C, Wang ZY, Dendukuri N, Lawn SD, Denkinger CM, Steingart KR. Lateral flow urine lipoarabinomannan assay for detecting active tuberculosis in HIV-positive adults. Cochrane Database of Syst Rev. 2016(5):CD011420. 10.1002/14651858.CD011420.pub2 PMC4916932 27163343

[21] Lawn SD, Kerkhoff AD, Vogt M, Wood R (2013). HIV-associated tuberculosis: relationship between disease severity and the sensitivity of new sputum-based and urine-based diagnostic assays. BMC Med..

[22] Nakiyingi L, Moodley VM, Manabe YC, Nicol MP, Holshouser M, Armstrong DT, Zemanay W, Sikhondze W, Mbabazi O, Nonyane BAS, Shah M, Joloba ML, Alland D, Ellner JJ, Dorman SE (2014). Diagnostic accuracy of a rapid urine lipoarabinomannan test for tuberculosis in HIV-infected adults. J Acquir Immune Defic Syndr..

[23] Lawn SD, Kerkhoff AD, Burton R, Meintjes G (2014). Underestimation of the incremental diagnostic yield of HIV-associated tuberculosis in studies of the Determine TB-LAM Ag urine assay. AIDS..

[24] Kerkhoff AD, Wood R, Vogt M, Lawn SD (2014). Predictive value of anemia for tuberculosis in HIV-infected patients in sub-Saharan Africa: an indication for routine microbiological investigation using new rapid assays. J Acquir Immune Defic Syndr..

[25] Talbot E, Munseri P, Teixeira P, Matee M, Bakari M, Lahey T, Reyn von F (2012). Test characteristics of urinary lipoarabinomannan and predictors of mortality among hospitalized HIV-infected tuberculosis suspects in Tanzania. PLoS One.

[26] Manabe YC, Nonyane BAS, Nakiyingi L, Mbabazi O, Lubega G, Shah M, Moulton LH, Joloba M, Ellner J, Dorman SE (2014). Point-of-care lateral flow assays for tuberculosis and cryptococcal antigenuria predict death in HIV infected adults in Uganda. PLoS One..

[27] Gupta-Wright A, Peters JA, Flach C, Lawn SD (2016). Detection of lipoarabinomannan (LAM) in urine is an independent predictor of mortality risk in patients receiving treatment for HIV-associated tuberculosis in sub-Saharan Africa: a systematic review and meta-analysis. BMC Med..

[28] Balcha TT, Winqvist N, Sturegård E, Skogmar S, Reepalu A, Jemal ZH, Tibesso G, Schön T, Björkman P (2014). Detection of lipoarabinomannan in urine for identification of active tuberculosis among HIV-positive adults in Ethiopian health centres. Trop Med Int Health..

[29] Kerkhoff AD, Wood R, Vogt M, Lawn SD (2014). Prognostic value of a quantitative analysis of lipoarabinomannan in urine from patients with HIV-associated tuberculosis. PLoS One..

[30] Peter JG, Zijenah LS, Chanda D, Clowes P, Lesosky M, Gina P, Mehta N, Calligaro G, Lombard CJ, Kadzirange G, Bandason T, Chansa A, Liusha N, Mangu C, Mtafya B, Msila H, Rachow A, Hoelscher M, Mwaba P, Theron G, Dheda K (2016). Effect on mortality of point-of-care, urine-based lipoarabinomannan testing to guide tuberculosis treatment initiation in HIV-positive hospital inpatients: a pragmatic, parallel-group, multicountry, open-label, randomised controlled trial. Lancet..

[31] Kerkhoff AD, Lawn SD (2016). A breakthrough urine-based diagnostic test for HIV-associated tuberculosis. Lancet..

[32] World Health Organization (2015). The use of lateral flow urine lipoarabinomannan assay (LF-LAM) for the diagnosis and screening of active tuberculosis in people living with HIV.

[33] Drain PK, Losina E, Coleman SM, Giddy J, Ross D, Katz JN, Walensky RP, Freedberg KA, Bassett IV (2014). Diagnostic accuracy of a point-of-care urine test for tuberculosis screening among newly-diagnosed HIV-infected adults: a prospective, clinic-based study. BMC Infect Dis..

[34] Nicol MP, Allen V, Workman L, Isaacs W, Munro J (2014). Urine lipoarabinomannan testing for diagnosis of pulmonary tuberculosis in children: a prospective study. Lancet Glob Health..

[35] Cannas A, Calvo L, Chiacchio T, Cuzzi G, Vanini V, Lauria FN (2010). IP-10 detection in urine is associated with lung diseases. BMC Infect Dis..

[36] Cannas A, Goletti D, Girardi E, Chiacchio T, Calvo L, Cuzzi G (2008). Mycobacterium tuberculosis DNA detection in soluble fraction of urine from pulmonary tuberculosis patients. Int J Tuberc Lung Dis..

[37] Petrone L, Cannas A, Vanini V, Cuzzi G, Aloi F, Nsubuga M (2016). Blood and urine inducible protein 10 as potential markers of disease activity. Int J Tuberc Lung Dis..

