# Supplementary material for: Diagnostic accuracy, incremental yield and prognostic value of Determine TB-LAM for routine diagnostic testing for tuberculosis in HIV-infected patients requiring acute hospital admission in South Africa: a prospective cohort
Source: BMC Med. 2017 Mar 21;15:67. doi: 10.1186/s12916-017-0822-8 (PMC5359871; doi:10.1186/s12916-017-0822-8)
Supplement: Additional file 1: Table S1. — Total clinical samples (‘research’ and ‘routine’ samples) sent for mycobacterial testing. (DOC 42 kb) [file 12916_2017_822_MOESM1_ESM.doc]

**Additional file 1: Table S1.** Total clinical samples (‘research’ and ‘routine’ samples) sent for mycobacterial testing.

| **Sample** | **Urine and sputum ‘research’ samples obtained in first 24 hours** | | **Total (research + routine) samples obtained during admission** | | | | |
| --- | --- | --- | --- | --- | --- | --- | --- |
|  | **No. (%) patients producing ≥ 1 sample** | **Total no. samples** | **No. (%) patients producing ≥ 1 sample** | **Total no. samples** | **Total no. culture and Xpert tests done** | **No. positive culture and Xpert tests (%)** | **No. (%) TB patients with ≥1 positive culture or Xpert test** |
| **SPUTUM** | 158 (37.0) | 279 | 245 (57.4) | 615 | 871 | 210 (24.1) | 75 (54.0)^a^ |
| **URINE** | 418 (97.9) | 418 | 418 (97.9) | 418 | 833 | 141 (16.9) | 89 (64.0) |
| **OTHER NON-RESPIRATORY SAMPLES** | - | - | 418 (97.9) | 712 | 687 | 91 (13.2) | 69 (49.6) |
| Ascitic fluid | - | - | 5 (1.2) | 5 | 5 | 1 (20.0) | 1 (0.7) |
| Blood | - | - | 410 (96.0) | 469 | 469 | 41 (8.7) | 41 (29.5) |
| Bone marrow | - | - | 2 (0.5) | 2 | 2 | 0 | 0 |
| Cerebrospinal fluid (CSF) | - | - | 76 (17.8) | 94 | 94 | 8 (8.5) | 8 (5.8) |
| Fine needle aspirate (FNA) | - | - | 19 (4.4) | 23 | 10 | 6 (60.0) | 6 (4.3) |
| Gastric lavage | - | - | 5 (1.2) | 7 | 7 | 2 (28.6) | 1 (0.7) |
| Pus | - | - | 5 (1.2) | 6 | 6 | 4 (66.7) | 3 (2.2) |
| Pleural fluid | - | - | 21 (4.9) | 29 | 29 | 17 (58.6) | 13 (9.4) |
| Stool | - | - | 9 (2.1) | 10 | 0 | 0 | 0 |
| Urine^b^ | - | - | 60 (14.1) | 63 | 61 | 11 (18.0) | 11 (7.9) |
| Other | - | - | 4 (0.9) | 4 | 4 | 1 (25.0) | 1 (0.7) |
|  |  |  |  |  |  |  |  |
| **TOTAL** | 420 (98.4) | 697 | 427 (100) | 1,745 | 2,391 | 442 (18.5) | 139 (100) |

^a^ Only 39 (28.1%) of total diagnoses could be made from sputum samples obtained in the first 24 hours.

^b^ Non-study urine samples sent for TB culture by the routine medical team later than the first 24 hour period.
