# Supplementary material for: Diagnostic accuracy, incremental yield and prognostic value of Determine TB-LAM for routine diagnostic testing for tuberculosis in HIV-infected patients requiring acute hospital admission in South Africa: a prospective cohort
Source: BMC Med. 2017 Mar 21;15:67. doi: 10.1186/s12916-017-0822-8 (PMC5359871; doi:10.1186/s12916-017-0822-8)
Supplement: Additional file 2: Table S2. — Diagnostic utility of urine-LAM by reference band cut-off and stratified by CD4 cell count. (DOCX 12 kb) [file 12916_2017_822_MOESM2_ESM.docx]

**Additional file 2: Table S2.** Diagnostic utility of urine-LAM by reference band cut-off and stratified by CD4 cell count.

|  | **Sensitivity**  **(%)** | **Specificity**  **(%)** | **Positive likelihood**  **ratio** | **Negative likelihood**  **ratio** | **Positive predictive**  **value (%)** | **Negative predictive**  **Value (%)** |
| --- | --- | --- | --- | --- | --- | --- |
| **LAM Grade 2 cut-off** | | | | | | |
| **Overall (n=413)** | 39.0 (30.7-47.7) | 98.9 (96.9-99.8) | 36.0 (11.5-113) | 0.6 (0.5-0.7) | 94.6 (85.1-98.9) | 76.8 (72.0-81.0) |
| CD4<100 (n=155) | 56.9 (44.7-68.6) | 97.6 (91.6-99.7) | 23.6 (5.9-94.3) | 0.4 (0.3-0.6) | 95.3 (84.2-99.4) | 72.3 (63.1-80.4) |
| CD4≥100 (n=258) | 18.8 (10.1-30.5) | 99.5 (97.2-100) | 36.4 (4.8-274) | 0.8 (0.7-0.9) | 92.3 (64.0-99.8) | 78.8 (73.1-83.7) |
| **LAM Grade 1 cut-off** | | | | | | |
| **Overall (n=413)** | 47.1 (38.4-55.8) | 95.3 (92.1-97.5) | 10.0 (5.7-17.6) | 0.6 (0.5-0.7) | 83.1 (72.9-90.7) | 78.6 (73.8-82.8) |
| CD4<100 (n=155) | 68.1 (56.0-78.6) | 92.8 (84.9-97.3) | 9.4 (4.3-20.7) | 0.3 (0.2-0.5) | 89.1 (77.8-95.9) | 77.0 (67.5-84.8) |
| CD4≥100 (n=258) | 23.4 (13.8-35.7) | 96.4 (92.7-98.5) | 6.5 (2.8-15.2) | 0.8(0.7-0.9) | 68.2 (45.1-86.1) | 79.2 (73.5-84.2) |
